# The chosen few: *Mycobacterium tuberculosis* isolates for IMPAc-TB

**DOI:** 10.3389/fimmu.2024.1427510

**Published:** 2024-10-28

**Authors:** Sasha E. Larsen, Hazem F. M. Abdelaal, Courtney R. Plumlee, Sara B. Cohen, Ho D. Kim, Holly W. Barrett, Qingyun Liu, Matthew H. Harband, Bryan J. Berube, Susan L. Baldwin, Sarah M. Fortune, Kevin B. Urdahl, Rhea N. Coler

**Affiliations:** ^1^ Seattle Children’s Research Institute, Center for Global Infectious Disease Research, Seattle Children’s, Seattle, WA, United States; ^2^ Department of Global Health, University of Washington, Seattle, WA, United States; ^3^ Department of Genetics, University of North Carolina at Chapel Hill, Chapel Hill, NC, United States; ^4^ Department of Immunology and Infectious Diseases, Harvard T. H. Chan School of Public Health, Boston, MA, United States; ^5^ Broad Institute of Massachusetts Institute of Technology (MIT), and Harvard, Cambridge, MA, United States; ^6^ Department of Immunology, University of Washington, Seattle, WA, United States; ^7^ Department of Pediatrics, University of Washington School of Medicine, Seattle, WA, United States

**Keywords:** *Mycobacterium tuberculosis*, tuberculosis, TB, lineages, diversity, isolates, IMPAc-TB

## Abstract

The three programs that make up the Immune Mechanisms of Protection Against *Mycobacterium tuberculosis* Centers (IMPAc-TB) had to prioritize and select strains to be leveraged for this work. The CASCADE team based at Seattle Children’s Research Institute are leveraging M.tb H37Rv, M.tb CDC1551, and M.tb SA161. The HI-IMPACT team based at Harvard T.H. Chan School of Public Health, Boston, have selected M.tb Erdman as well as a novel clinical isolate recently characterized during a longitudinal study in Peru. The PHOENIX team also based at Seattle Children’s Research Institute have selected M.tb HN878 and M.tb Erdman as their isolates of choice. Here, we describe original source isolation, genomic references, key virulence characteristics, and relevant tools that make these isolates attractive for use. The global context for M.tb lineage 2 and 4 selection is reviewed including what is known about their relative abundance and acquisition of drug resistance. Host–pathogen interactions seem driven by genomic differences on each side, and these play an important role in pathogenesis and immunity. The few M.tb strains chosen for this work do not reflect the vast genomic diversity within this species. They do, however, provide specific virulence, pathology, and growth kinetics of interest to the consortium. The strains selected should not be considered as “representative” of the growing available array of M.tb isolates, but rather tools that are being used to address key outstanding questions in the field.

## 1 Introduction

Across each of the Immune Mechanisms of Protection Against *Mycobacterium tuberculosis* Centers (IMPAc-TB), there are mechanistic questions of special focus. The CASCADE team at Seattle Children’s Research Institute are examining the stages of infection through detailed interrogation of pulmonary pathology and natural or vaccine-induced protection across this continuum in mice, non-human primates, and human studies. The HI-IMPACT team based at Harvard T.H. Chan School of Public Health, Boston, are leveraging key immune correlates of protection from their seminal intravenous (IV) bacillus Calmette–Guérin (BCG) vaccination strategy in rhesus macaques (1, 2) and comparing those with human cohort responses with computational modeling to better design vaccines against tuberculosis (TB). The PHOENIX team based at Seattle Children’s Research Institute are focused on a cross-species analysis of candidate vaccine-induced immunity and efficacy for prevention of disease or infection in preclinical mouse, guinea pig, and non-human primates as well as a human aerosol challenge model using BCG as a representative mycobacteria.

While these centers are focused on discrete but complementary work, each are using modeling, escalating from *in vitro* or *in silico* through *in vivo* preclinical and first-in-man experimental medicine studies. In the TB research field, we challenge *in vitro* or *in vivo* models with “representative” isolates of *Mycobacterium tuberculosis* (M.tb) that fit some categorical aspect of what we would like to examine or that provide continuity with previous work to continue a specific story. Recent data are challenging the use of “representative” to define our M.tb isolates of choice and instead suggest there is an underrepresentation of the breadth of biological diversity that exists in M.tb (3–5). Indeed, even preclinical drug therapy studies have demonstrated significant differences in *in vivo* sensitivity between M.tb strains (6). Next-generation tools and use of big data pipelines may allow us to better profile huge arrays of M.tb isolates or lineages in the near future, as groups within this consortium are already leveraging artificial intelligence and systems biology for complex data and predictive modeling.

In the absence of current or established tools that allow us to readily select challenge isolates spanning a vast array of M.tb lineages, it was necessary for each of the IMPAc-TB consortiums to choose the few they would study across their programs. The six strains used in the CASCADE (SCRI), HI-IMPACT (Harvard), or PHOENIX (SCRI) centers are described here (**Table 1**). We outline the source of each isolate, reference the sequenced genome if available, include specific tools in that background, give a summary of models using that strain, and provide seminal aspects of the isolate including virulence or kinetics if known and key historical work using that isolate. These basic features helped define the selection of isolates for specific consortium endpoints.

**Table 1:** M.tb isolates being used across the three IMPAc-TB consortiums.

Program	Lineage 2	Lineage 4
CASCADE	SA161	H37Rv, CDC1551
HI-IMPACT	Clinical Peru Isolate	Erdman
PHOENIX	HN878	Erdman

## 2 Materials and methods

### 2.1 M.tb genome sequencing

#### 2.1.1 Genomic DNA extraction

In order to obtain high-quality genomic DNA (gDNA) from M.tb cultures of all of the isolates except the g2g clinical isolate described above, we followed the methodology described previously (7). In summary, mycobacterial cultures (M.tb SA161, H37Rv, CDC1551, Erdman, and HN878) were grown to an optical density 600 nm (O.D. 600) of 1.5. Subsequently, the cultures were harvested by centrifugation, washed in PBS, and dissolved in TE buffer. To lyse the mycobacteria, the bacterial suspension was subjected to 80°C for 20 min. After allowing the tubes to cool to room temperature, lysozyme solution (100 mg/ml) was added to each tube, followed by incubation at 37°C for 3 h with intermittent mixing. Next, a mixture of SDS (10%) and proteinase K (20 mg/ml) was added to each tube in an 88:12 ratio. The tubes were then incubated at 65°C for 2 h. Following this incubation period, 100 µl of 5 M NaCl was added to each tube and incubated at 65°C for an additional 10 min. Subsequently, an 80 µl aliquot of 10% cetyl trimethyl ammonium bromide (CTAB) solution (Sigma-Aldrich, St. Louis, MO) was added, mixed thoroughly, and incubated at 65°C for another 10 min. The DNA was then extracted first with an equal volume of phenol/chloroform/isoamyl alcohol (25:24:1 v/v/v) and then with chloroform/isoamyl alcohol (24:1 v/v). The DNA was precipitated by adding 0.6 volumes of ice-cold isopropanol. The precipitated DNA was pelleted by centrifugation at 10,000 rpm and 4°C for 15 min. The resulting DNA pellets were washed with cold 75% ethanol and dried using a SpeedVac for 5 min. Finally, the genomic DNA was resuspended in 50 µl of sterile distilled water. The quality of the gDNA was assessed using a NanoDrop spectrophotometer (Thermo Scientific, Wilmington, DE).

#### 2.1.2 Library production

Starting with a minimum of 750 ng of DNA, samples are sheared in a 96-well format using a Covaris LE220 focused ultrasonicator targeting 380-bp inserts. This insert size improves overall library performance and allows the longer sequencing read lengths on Illumina sequencing platforms (150 bp) to be efficiently used without producing a significant number of overlapping reads. The resulting sheared DNA was cleaned with Agencourt AMPure XP beads to remove sample impurities prior to library construction. Shearing was followed by size selection performed using the KAPA Hyper Prep kit (KR0961 v1.14). End-repair, A-tailing, and ligation are performed as directed. Two final AMPure cleanups are performed after ligation to remove excess adapter dimers from the library. All library construction steps are automated on the Perkin Elmer Janus platform. Library yield was quantified using Quant-IT dsDNA High Sensitivity kit (Invitrogen Q33120). Libraries were validated in triplicate using the Bio-Rad CFX384 Real-Time System and KAPA Library Quantification Kit (KK4824).

#### 2.1.3 Whole-genome sequencing

Massively parallel sequencing-by-synthesis with fluorescently labeled, reversibly terminating nucleotides was carried out using the Illumina NovaSeq 6000 platform (RTA 3.1.5) and was performed at the University of Washington Center for Rare Disease Research (UW-CRDR). Barcoded genome libraries were pooled with liquid handling robotics prior to loading. Raw sequence reads with an average read length of 150 bp were assembled against the sequence of the reference strain M.tb H37Rv (NCBI accession number: NC_018143.2) using SPADES v3.15.4 (8). Whole-genome sequences were deposited into GenBank BioProject: PRJNA1051793, as described with specific accession numbers stated.

### 2.2 *In vivo* mouse challenge and survival studies

In all studies, mice were housed in the Seattle Children’s Research Institute (SCRI) biosafety level 3 (BSL-3) animal facility under pathogen-free conditions and were handled in accordance with approved protocols from the SCRI Institutional Animal Care and Use Committee (IACUC). All methods were carried out in accordance with animal welfare guidelines and regulations. For survival studies, breeding pairs of C57BL/6 bg/bg (beige) mice were originally purchased through Jackson Laboratories. In-house bred male beige mice were used for experiments at 4–6 weeks of age and had not been previously used for breeding purposes. Mice were maintained at four animals per cage. Mice were challenged with a low-dose aerosol (target 25–100 CFU per mouse upon infection) of either M.tb SA161 or M.tb HN878 using a Glas-Col whole-body aerosol infection chamber. Bacterial burden was evaluated at 24 h and 4 weeks post challenge as previously described (9). Survival was monitored in 10 mice per group following M.tb infection. Animals with greater than 20% weight loss, or moribund condition, were euthanized. For ultra-low dose aerosol (targets 1–3 CFU per mouse upon infection) studies, female C57BL/6 mice purchased from Jackson Laboratories (age 8–27 weeks old) were challenged with either M.tb Erdman or M.tb H37Rv also using a Glas-Col whole-body aerosol infection chamber. Pulmonary bacterial burden was evaluated 35–42 days post challenge.

### 2.3 Statistical analysis

For *ex vivo* plating of organ homogenate to enumerate bacterial CFU in a single organ and timepoint (24 h, 4 weeks, or 6 weeks post-challenge), we used a two-tailed unpaired Student’s t test to compare between M.tb challenge strains. For probability of survival, cohorts were compared using the log-rank Mantel–Cox test. All statistical analysis was performed using GraphPad Prism version 10.2.2.

## 3 Results

### 3.1 M.tb isolates

As the consortium matures, we are reflecting on opportunities to cross-validate or harmonize so the discrete but complimentary data being generated can have the largest impact for the TB pathogenesis, immunology, and vaccine communities. Interestingly, and without prior discussions, each of the programs focused their M.tb isolates across two modern lineages, 2 and 4. Lineage 2 is notable in its expanding global distribution, “hypervirulence,” correlations with relapse, and enriched acquisition of antibiotic drug resistance (10–16). *In vitro*, lineage 2 Beijing isolates persist and grow normally within macrophages compared with other lineages that are relatively more inhibited once they are intracellular (17). These features make lineage 2 high-priority in preclinical studies for evaluation of disease progression and efficacy of vaccine candidates. M.tb isolates SA161 and HN878 and the clinical isolate from Peru are lineage 2 isolates represented in the IMPAc-TB consortiums. Lineage 4, the Euro-American lineage, is also well distributed globally and can be associated with unique features of disease pathology and treatment failure in humans (18–21). M.tb H37Rv, Erdman, and CDC1551 are each lineage 4 isolates being leveraged by one or more of the IMPAc-TB programs. Each isolates’ key features and rationale for selection are described below.

### 3.2 M.tb HN878 (lineage 2)

M.tb HN878, a W-Beijing lineage isolate, was sourced by the PHOENIX consortium directly from BEI Resources (NR-13647). This clinical isolate was derived from a patient sample in Houston, Texas, in a 1990s outbreak and has been well studied since (22) with the parental genome sequence available on GenBank: ADNF01000000.1. Many other M.tb-derived materials including M.tb HN878 whole-cell lysate (NR-14824), derived lipid fraction (NR-14839), and genomic DNA (NR-14867) are available within the BEI Resources Repository. Defined as hypervirulent (23), M.tb HN878 induces a waning host T helper 1 (Th1) response and rapid expansion of regulatory T cells in chronic preclinical settings (22), as well as progressive pulmonary pathology and morbidity in C57BL/6 mice (24, 25). Interestingly, this isolate can acquire large genetic duplications with serial *in vitro* passage dramatically reducing virulence *in vivo (* 25, 26). For the PHOENIX consortium, we generated a large batch single passage culture in 2019 to avoid the duplication issue and ensure continuity across studies, and we confirmed virulence of our stock culture in mouse survival studies (25). The single passage PHOENIX batch of M.tb HN878 was sequenced, and that genome can be found at GenBank: SAMN38797941.

The M.tb HN878 strain has been evaluated extensively *in vitro* as well as in preclinical mouse and guinea pig models of aerosol challenge. This includes recent examinations of single-cell preparations and of the influence of detergents on the mycobacterial cell wall and their impacts on interactions with innate immune cells (27). *In vivo*, M.tb HN878 induces more granulomatous-like structures in preclinical models that better reflect human pathology than other well-used isolates (22, 23, 28). This includes induction of a blood-based TB transcriptional signature in C3HeB/FeJ mice by M.tb HN878 infection, whereas similar studies using less virulent M.tb H37Rv to infect C57BL/6 mice did not result in production of this human-derived biomarker of TB disease (29). Recent evidence suggests that while aerosol infection with a high dose of M.tb HN878 does induce bone-marrow-derived myelopoiesis, there is a concomitant reduction in trained innate immune responses in a mouse model (30). A key feature which led to the selection of this strain is that the high virulence of M.tb HN878 may be due to the preferential recruitment of pathology-inducing neutrophils and relatively damped adaptive responses (29). Despite the observed virulence and immune skewing from clinical M.tb HN878 isolate infection, prophylactic vaccination with BCG provides protection from bacterial burden and pulmonary pathology and enhances survival in several preclinical models (31–33). M.tb HN878 was selected by the PHOENIX consortium due to its well profiled virulence discussed above and its membership in lineage 2, which has high global prevalence. This M.tb isolate serves as a suitable strain to evaluate vaccine-specific efficacy in both prevention of infection (POI) and prevention of disease (POD) contexts, key components of the PHOENIX goals for the program. To our knowledge, this will also be the first time M.tb HN878 is used as a challenge strain in nonhuman primates.

### 3.3 M.tb SA161 (lineage 2)

M.tb SA161 is also a W-Beijing-type Lineage 2 isolate (34). SA161 is derived from a cluster of cases in Arkansas and was originally sourced by the CASCADE team from Dr. Ian Orme at Colorado State University (34). The M.tb SA161 being used by the Urdahl Lab in the CASCADE program was sequenced, and the genome sequence is deposited at GenBank: SAMN38797942.

Like M.tb HN878, SA161 is a highly virulent W-Beijing strain of M.tb (35). Historical data suggest that lineage 2 Beijing isolates may associate with TB infections in persons living with human immunodeficiency virus (PLWHIV) (35). As such, M.tb SA161 has been evaluated in several models with seminal features of HIV infection including mice transgenic for HIV transcription protein Tat (35). M.tb SA161 is frequently paired with highly susceptible mouse strains like C3HeB/FeJ to model high frequencies of pulmonary pathology and use survival as primary endpoints (32, 35). Despite the highly virulent M.tb—highly susceptible mouse strain pairing—prophylactic vaccination with BCG still affords protection from bacterial burden and pathology in both mouse and guinea pig infections at intermediate timepoints (32, 36). At later timepoints post challenge, W-Beijing strains M.tb HN878 and M.tb SA161 upregulate regulatory T-cell responses and the early protection wanes as mice succumb to high bacterial burden (> 6 Log10 CFU) and pathology in a more resistant C57BL/6 mouse strain (31), which was a key feature for its selection in this consortium.

**Figure 1:**
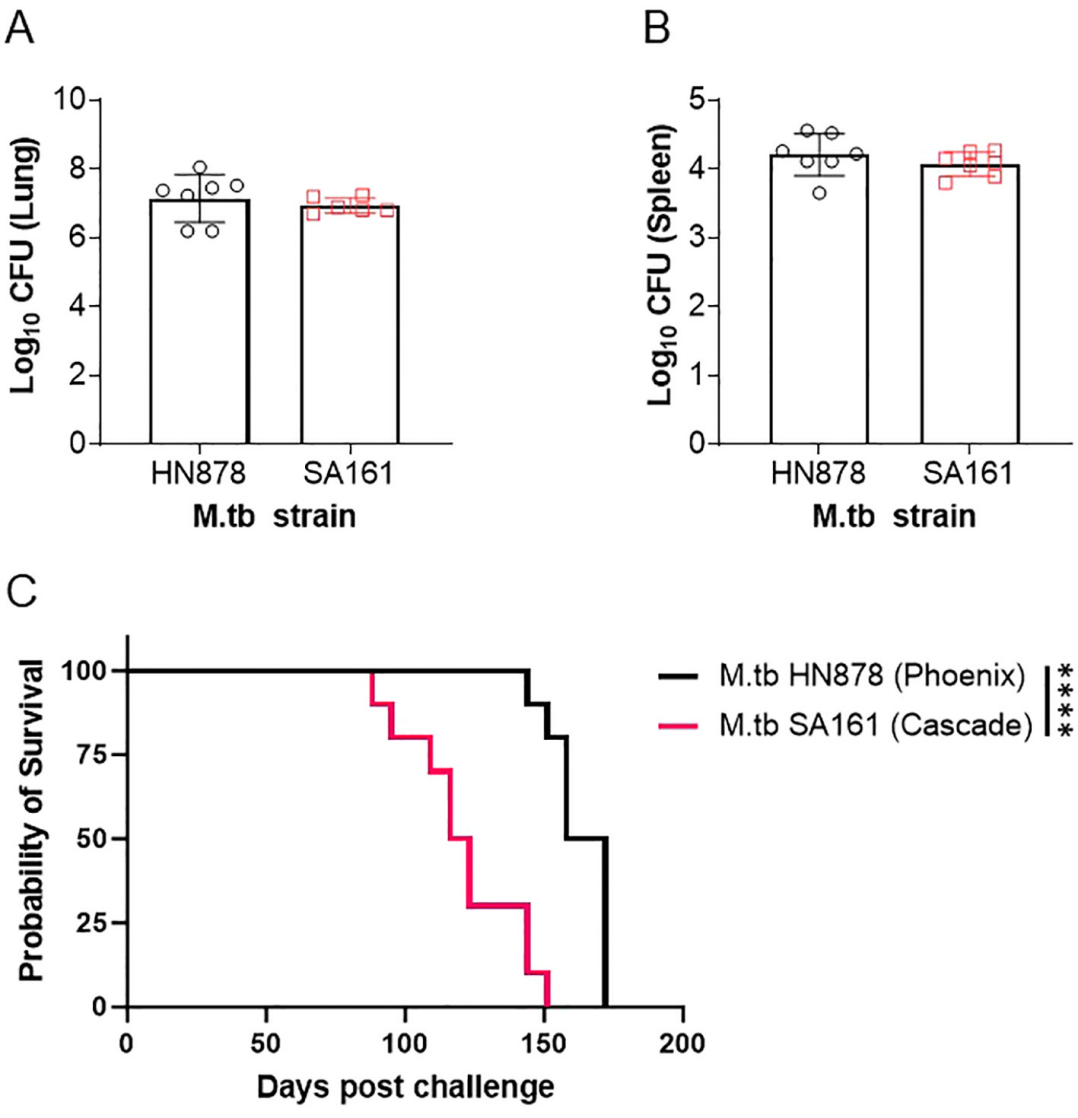
Survival of mice challenged with Beijing isolates of M.tb. Male Beige mice were challenged with a low-dose aerosol of either M.tb HN878 (open black circles) or M.tb SA161 (open red squares). At 4 weeks post-challenge, **(A)** lung and **(B)** spleen bacterial burden were evaluated in n=6-7. Two-tailed unpaired Student’s t test found no significant differences between lung (p = 0.4997) or spleen (p = 0.3365) groups. Mean Log_10_ CFU for lung = 7.1 HN878 and 6.9 for SA161. Mean Log_10_ CFU for spleen = 4.2 HN878 and 4.0 for SA161 **(C)** Cohorts of = 10 mice per challenge (M.tb HN878 = black line or M.tb SA161 = red line) isolate were followed over time with weekly or more frequent weighing as a measure of morbidity due to infection. When mice reached 20% weight loss, they were considered moribund and humanely euthanized. Asterisks represent significance (p < 0.0001) between the groups using a Log-rank Mantel–Cox test comparing survival curves. Data representative of one experiment.

At the start of the IMPAc-TB contract, the PHOENIX and CASCADE consortiums performed a head-to-head virulence survival study of M.tb SA161 and M.tb HN878 to both compare their relative virulence as lineage 2 members as well as infer any similarities or differences worth noting for future comparisons across programs. In this study, male Beige mice were challenged with a low-dose aerosol (25–100 CFU) of either lineage 2 isolate. Beige mice were used in this study because they are highly susceptible to mycobacterial infections and offered a compressed survival timeline (<200 days) for this exploratory head-to-head study. In addition, both in human epidemiology and in mouse preclinical studies, males are more sensitive to TB disease [well reviewed here (37)], which also afforded an accelerated assessment between M.tb isolates. Samples were collected 24 h post challenge to confirm deposition in lungs, and for HN878, we observed 23, 67, and 52 CFU per mouse (averaging 47 CFU) and 14, 31, and 59 CFU were observed for SA161 challenge (average 35 CFU; n=3 per group). At 4 weeks post challenge, lung and spleen bacterial burden were evaluated (n=6–7) with no significant difference in either site between the two challenge strains at this timepoint observed (**Figures 1A, B**). A cohort of mice (n=10 each) was followed for morbidity endpoints resulting in probability of survival over time (**Figure 1C**). The median survival for mice challenged with M.tb HN878 was 165 days, whereas the median survival for M.tb SA161 infected mice was 119.5 days.

In this comparative study, we observed a different survival profile in a Beige mouse background between M.tb HN878 and M.tb SA161 low-dose aerosol challenges, despite relatively equivalent bacterial burden at 4 week post challenge in both the lung and spleen. Interestingly, M.tb SA161 induces an earlier initiation of morbidity (day 88 to 151, 63 days of group decline) but the overall group mortality is more heterogeneous over time when compared with M.tb HN878 (day 144 to 172, 28 days of group decline), which seems to have a relatively delayed but more uniform morbidity of the total group. To our knowledge, this is the first report in male Beige mice for either M.tb isolate with these endpoints. This different pattern of morbidity between isolates in highly sensitive Beige mice mirrors that observed in the literature in more resistant C57BL/6 mice (31); however, the data reported here are less virulent in Beige mice comparatively by survival presumably due to the lower challenge dose at infection. In the context of the PHOENIX program where differences between vaccine groups are being evaluated, M.tb HN878 is a preferred isolate, but if, in contrast, interest is in the mechanism of disease progression and host variability in progressions to disease, then M.tb SA161 is a more reasonable strain to select. In the context of the CASCADE program, M.tb SA161 has been the primary isolate used to characterize pathological and immunological disease outcomes following vaccination due to its ability to generate large necrotic granulomas in the C3HeB/FeJ background. These profiles and specific endpoints of interest are well tailored to the overall objectives of the PHOENIX and CASCADE programs.

### 3.4 Clinical Peru isolate (lineage 2)

A distinct clinical isolate was recently discovered after the initiation of the IMPAc-TB program during a genome-to-genome (G2G) study conducted in Lima, Peru (38) (manuscript accepted, pending publication, SRA accession codes referenced there). This G2G study was designed to identify the genetic interaction between host genetic background and M.tb genetic background. The researchers discovered a single host variant (located in a non-coding region of the FLOT1 gene) that was associated with infection by a particular M.tb strain they called g2g-L2. They found the g2g-L2 strain locally emerged in Peru around 60 years ago but has since undergone rapid expansion. In a 2010 cohort, the g2g-L2 strain accounted for 6.6% of all M.tb strains circulating in Lima, Peru, but by 2020, the prevalence had almost doubled (12.6%). Additionally, it was shown that 87% of the g2g-L2 strains were in genomic clusters, whereas the clustering rates for the neighboring L2 clades were only 18% to 28%. These data indicate that the g2g-L2 strain has increased transmissibility in the local population. While the g2g-L2 strain can transmit successfully in Lima, Peru, it was not found elsewhere in the world. The authors also showed that g2g-L2 strains have a distinctive redox state compared with their nearest neighbor strains, manifested as a more oxidative cell state and resistance to reductive stress. The authors narrowed down a mutation (Thr2Asn) in trxB2 that was specific to the g2g-L2 strain, and they found that this trxB2 mutation can lead to higher activity of thioredoxin reductase and a significant shift in the NAD+/NADH ratio toward the oxidized state.

The authors delved into the underlying interaction between Mtb strains (g2g-L2 vs. nearest-neighbor strains, termed non-g2g-L2) and host immune cells with differing genetic backgrounds (FLOT1, AT vs. TT). RNA-seq analysis was employed to profile the innate immune response to M.tb infection, with the expression of 20 infection-responsive genes used to gauge the “infection score,” where higher expression indicated a stronger response. The study revealed that overall, AT host cells exhibited an elevated infection response compared with TT host cells. However, within the AT host cells, g2g-L2 infection blunted this response relative to non-g2g-L2 infection. Specifically, FLOT1-AT cells exhibited increased expression of genes associated with type 1 and type 2 interferon pro-inflammatory signaling, MHC-I antigen processing and presentation, IL-1B signaling, cytosolic DNA sensing, and zinc homeostasis. The authors posited that FLOT1-AT donors mount a robust transcriptional response to infection, potentially due to increased sensitivity in inducing innate responses. However, this response was skewed toward interferon following non-g2g-L2 infection, contrasting with IL-1B dominant responses observed after g2g-L2 infection. As the most recently isolated M.tb within the consortium, this g2g strain helps ground the clinical relevance of findings back to human epidemiologic data, a feature unique to the HI-IMPACT program.

### 3.5 M.tb Erdman (lineage 4)

M.tb Erdman K01 was sourced by the PHOENIX consortium directly from BEI Resources (NR-15404). This original clinical isolate was derived from human sputum at the Mayo Clinic in 1945 and is noted to be highly virulent (39). A low passage culture of M.tb Erdman was generated by the Coler Lab in the PHOENIX program. The stock material was sequenced and the assembled genome deposited at GenBank: SAMN38797939. A notable feature of M.tb Erdman was the development of a barcoded library (BEI Resources NR-50781) where each bacterium contains a unique and trackable sequence, enabling the profiling of a single cells’ longitudinal fate *in vivo* . This library has been leveraged in non-human primate models coupled with pathology and imaging to interrogate individual lesion-level disease progression and infection dynamics (40).

M.tb Erdman has been extensively used for aerosol challenge with diversity outbred mice to identify critical gene loci (41–44), innate cell influx, pulmonary necrosis (45), and age-related influences (46) that correlate with a spectrum of disease in this model. In mice, prophylactic BCG vaccination provides protection from early pulmonary and peripheral bacterial burden after challenge with M.tb Erdman (33). Studies in cynomolgus macaques infected with M.tb Erdman identified peripheral blood transcriptional changes occurring early (<6 months) post infection (47), which share similarities with human risk signatures predicting advancement to active TB disease. The similarities in risk signatures between M.tb Erdman-infected macaques and humans highlights the usefulness of this model to predict vaccine outcomes in humans.

### 3.6 M.tb H37Rv (lineage 4)

M.tb H37 was isolated from a human lung sample at the Trudeau Laboratory in 1905. M.tb H37Rv was derived from this isolate after selection for rough colony morphology and virulence in 1934 (48) and is one of the most widely used strains of M.tb available. Despite researchers identifying notable polymorphisms between M.tb H37Rv isolates maintained in different laboratories (49), the M.tb H37Rv genome is commonly used as the base reference. Indeed, antigens included in high-priority clinical vaccine candidates are sourced from the H37Rv genome, including ID93 (50), M72 (51), GamTBvac (52), and H107e (53). The M.tb H37Rv being leveraged by the CASCADE consortium was a gift from the lab of Dr. Joel Ernst. This isolate was derived from ATCC TMC 102 (catalog #27294), and the genome was partially sequenced and published in 1998. Despite this isolate serving as the *de facto* M.tb genome reference, the full genome sequence was only recently completed, comprising over 6,000 novel base pair regions, using a new paradigm of assembly called Bact-Builder (54). The CASCADE program M.tb H37Rv strain has been serially passaged through mice and has maintained virulence properties compared with other lab-adapted strains, perhaps through retention of the virulence factor PDIM, which is rapidly lost upon *in vitro* passage (55). The CASCADE M.tb H37Rv isolate was sequenced, and that assembled genome can be found at GenBank: SAMN38797937.

**Figure 2:**
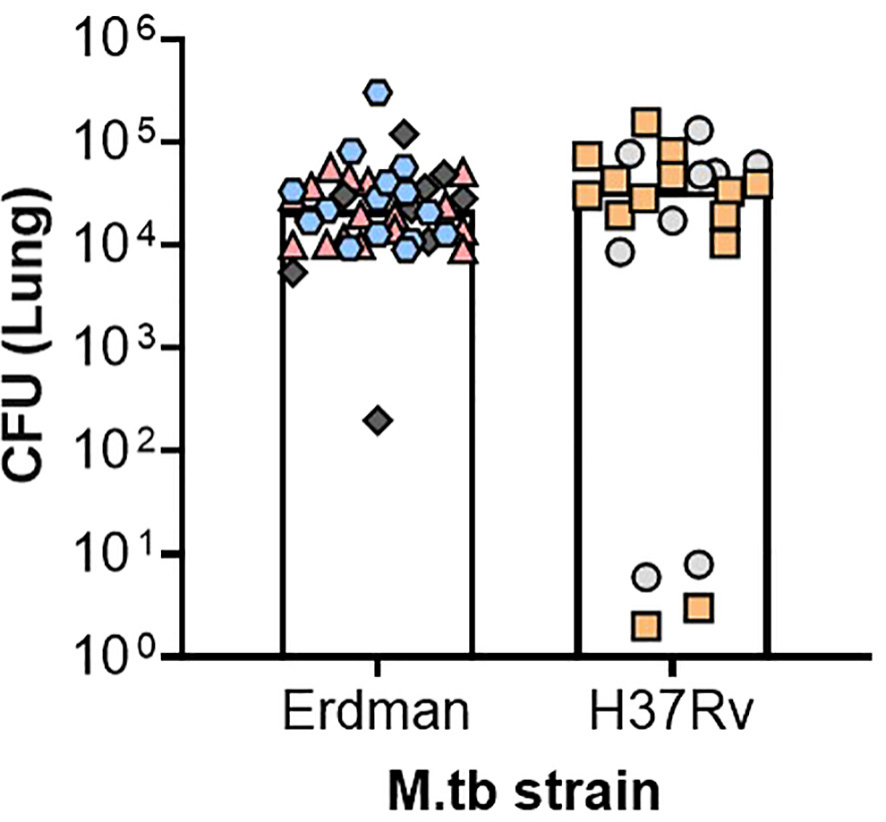
Comparable pulmonary bacterial burden between M.tb H37Rv and Erdman after ultra-low-dose challenge. Female C57BL/6 mice were challenged between 8 and 27 weeks of age with an ultra-low-dose challenge of either M.tb Erdman or M.tb H37Rv. At 35–42 days post-infection, lung bacterial burden was evaluated. Data depict six different challenge experiments, denoted in the figure as different-colored shapes, where each shape represents an individual mouse whose CFU was over 1. Two-tailed unpaired Student’s t test found no significant differences between the challenged groups (p = 0.5196) where the mean CFU for M.tb Erdman was 3.4 × 10^4^ and for M.tb H37Rv was 4.2 × 10^4^.

Like M.tb Erdman, M.tb H37Rv has been developed as a barcoded strain, allowing for the individual tracking and monitoring of deposited bacteria at the time of challenge and through chronic timepoints of infection and disease (56). This is an especially useful tool in the context of ultra-low-dose aerosol infections where as few as one bacterium per mouse is inhaled and monitored over time (56), better reflecting the conditions of human challenge. In general, M.tb H37Rv laboratory isolates are notably less virulent across models, with higher rates of survival and less necrotic lesions in the translational guinea pig preclinical model compared with other strains being used in this program including M.tb Erdman, M.tb HN878, or M.tb CDC1551 (23). Lower virulence of M.tb H37Rv compared with M.tb Erdman has also been observed in a New Zealand White rabbit model of aerosol challenge by 5 weeks post infection (57). Despite these reports, the CASCADE M.tb H37Rv isolate has similar virulence to M.tb Erdman and is more virulent than M.tb CDC1551, in studies by the group in C57BL/6 mice (**Figure 2** and article by some of these same authors presented in this special issue (58), respectively). These patterns of virulence align with prior studies using the CASCADE programs’ M.tb H37Rv in C57BL/6 mice (56, 59). As such, the CASCADE program has used H37Rv as the primary lineage 4 strain to draw direct comparisons with M.tb SA161. Interestingly, in a BALB/c drug therapy model, different isolates of M.tb H37Rv were similarly sensitive to front-line drug treatment with rifampicin, isoniazid, and pyrazinamide as M.tb Erdman (6). However, M.tb H37Rv has been the strain of choice to date when assessing genetic components influencing host susceptibility in the context of the collaborative cross mice (60–63).

### 3.7 M.tb CDC1551 (lineage 4)

M.tb CDC1551 is a clinical isolate collected in the mid-1990s amid an outbreak with uncharacteristically highly positive tuberculin skin testing rates in rural Kentucky and Tennessee (64). The CASCADE M.tb CDC1551 isolate, originally a gift from W.R. Bishai, was sequenced, and that assembled genome can be found at GenBank: SAMN38797940. The BEI repository hosts numerous M.tb CDC1551-derived materials including cell lysate (NR-14835), polyclonal anti-sera from a guinea pig infection (NR-13818), genomic DNA (NR-48981), and a large assortment of transposon mutants. Live-attenuated vaccines are gaining traction in the TB research community, and one notable vaccine based in a parent CD1551 background is M.tbΔsigH (65).

M.tb H37Rv, M.tb Erdman, and M.tb CDC1551 contain a frame-shift deletion in a gene cluster responsible for phenolic glycolipid (PGL) production, resulting in a relatively reduced virulence compared with lineage 2 isolates (including M.tb HN878) which do not contain this deletion (66). Furthermore, prophylactic BCG vaccination provides better protection against these lineage 4 isolates compared with lineage 2 isolates in an intravenous infectious challenge of pooled barcoded M.tb in a C57BL/6 mouse model (67). Although the initial paper describing this strain in an *in vivo* mouse model found that, when compared with M.tb Erdman, M.tb CDC1551 expanded rapidly and notably reached two logs higher lung bacterial burden by day 20 (64), curiously, the M.tb Erdman peaked at ~10^4^, which is ~2 logs lower than the typical CFU load following infection with virulent M.tb strains, such as M.tb Erdman or M.tb H37Rv. A subsequent study found that M.tb CDC1551 was actually less virulent than several other M.tb strains tested, including M.tb H37Rv, M.tb Erdman, and M.tb HN878, while inducing a more hyperinflammatory immune response (68). Consistent with the more recent study, the CASCADE M.tb CDC1551 isolate is less virulent in C57BL/6 mice than the CASCADE M.tb H37Rv isolate, with an approximately 1-log reduction in the peak lung bacterial burden.

## 4 Discussion

Aside from strain identity and genome sequence, there are many factors that influence how M.tb can behave both *in vitro* and *in vivo*. This includes culture conditions like the presence, absence, or concentration of Tween which directly changes the cell wall and some subsequent interactions with host cells (69–71) or growth medium albumin choice. Large first stock batches are made for each consortium where M.tb were grown in 10% OADC, 0.05% Tween, and in the absence of C0_2_. For enumeration for mouse infection studies, the CASCADE program uses OD600nm of 1.0 and Phoenix uses direct CFU plating of aliquots and both programs combine these results with empirical titration experiments to determine nebulizer stock dilutions to attain the infection range per mouse desired. The Coler, Fortune, and Urdahl labs serve as distributor partners for each consortium where large low-passage batches (>900 1.0-ml aliquots in the case of the PHOENIX program) are made and stocks are distributed to contract partners to reduce the influence of strain passage on results obtained within each consortium. Annual CFU plating by all partners is a contract deliverable for some of the consortiums as a simple and direct check that stocks remain viable and at or near expected CFU. The status of specific lipids, like phthiocerol dimycocerosates (PDIMs) on the bacterial cell envelope, which have been observed to affect pathogenesis and virulence, is a similar factor important for understanding and comparing M.tb strain outcomes (72). The mouse-passaged M.tb H37Rv used by the CASCADE program recently confirmed PDIM status (73), and M.tb Erdman was similarly confirmed prior to DNA barcoding (40) and no subsequent colony purification occurred. PDIM status was not a planned screen throughout the program, although we acknowledge that this could affect results.

While each program is using different isolates, they are consistently from lineages 2 and 4, which provides some harmony of interest. Importantly, each isolate suits the needs of each consortium; for example, CASCADE is mechanistically interrogating the diversity of the immune response including pathology endpoints and the variability within an M.tb SA161 infection, possibly identifying key differences in disease progression. Conversely, PHOENIX is interested in vaccine efficacy endpoints and M.tb HN878 provides a more homologous response and disease progression that affords resolution between immunization strategies. The ability of HI-IMPACT to leverage a recent clinical isolate with a direct relationship to epidemiological data and human genetic variabilities make their work in preclinical models highly translational and informative. However, this collective work has excluded other lineages, including 1 and 3 which are found in Southeast Asian countries like Indonesia and the Philippines, which are among the top eight high-TB burden countries globally (74). Should these consortiums find critical disparities in relative immune responses or protection from vaccinations, it would be necessary to perform similar analyses using lineages 1 and 3. Other *ex vivo* tools like the mycobacterial growth inhibition assay (75–77), which allow for multiple challenge isolates to be used, are also being integrated into specific programs and may help address this need for an increased breadth of M.tb challenges to increase the scope of findings and areas of follow-up.

By identifying salient differences between strains being used across the three programs, we aim to transparently identify what may be common findings versus those that may be unique due to model organism selection. It is clear from the literature that whole-genome sequencing can reveal key genes involved in drug resistance, adaptations to stress conditions, and virulence of M.tb isolates (78–80). The sequences reported here aim to contribute to those efforts and provide pathogen genomic comparators for phenotypic outcomes across the consortium. Future work will align the sequences reported here to parent lineage-specific reference genomes available, clinical isolates, and an ancestral, non-lineage-specific, M.tb complex genome (termed MTBC_0_) (81). We expect that placing these isolates in the advanced phylogeny available for M.tb, through a number of platforms such as TB-Annotator (82) or MAGMA (83), will provide more opportunities to further link protection or pathogenic outcomes derived from IMPAc-TB with existing literature and clinical trends. In addition to M.tb isolate identity, the consortium is actively discussing challenge doses and how this directly influences outcomes in the preclinical models being studied. Furthermore, use of diversity outbred and collaborative cross mouse strains is an acknowledgement that host diversity plays a significant role in TB outcomes and may help uncover critical immune correlates of immunity.

## Data Availability

The datasets presented in this study can be found in online repositories. The names of the repository/repositories and accession number(s) can be found below: https://www.ncbi.nlm.nih.gov/genbank/, BioProject: PRJNA1051793.
